# Contactless In-Home Monitoring of the Long-Term Respiratory and Behavioral Phenotypes in Older Adults With COVID-19: A Case Series

**DOI:** 10.3389/fpsyt.2021.754169

**Published:** 2021-10-27

**Authors:** Guo Zhang, Ipsit V. Vahia, Yingcheng Liu, Yuzhe Yang, Rose May, Hailey V. Cray, William McGrory, Dina Katabi

**Affiliations:** ^1^Computer Science and Artificial Intelligence Laboratory, Massachusetts Institute of Technology, Cambridge, MA, United States; ^2^Division of Geriatric Psychiatry, McLean Hospital, Belmont, MA, United States; ^3^Department of Psychiatry, Harvard Medical School, Boston, MA, United States; ^4^Mary Ann Morse at Heritage Affordable Senior Living, Framingham, MA, United States

**Keywords:** COVID-19, long-term outcomes, phenotypes, contactless monitoring, respiration, behavior, older adults, case report

## Abstract

Currently, there is a limited understanding of long-term outcomes of COVID-19, and a need for in-home measurements of patients through the whole course of their disease. We study a novel approach for monitoring the long-term trajectories of respiratory and behavioral symptoms of COVID-19 patients at home. We use a sensor that analyzes the radio signals in the room to infer patients' respiration, sleep and activities in a passive and contactless manner. We report the results of continuous monitoring of three residents of an assisted living facility for 3 months, through the course of their disease and subsequent recovery. In total, we collected 4,358 measurements of gait speed, 294 nights of sleep, and 3,056 h of respiration. The data shows differences in the respiration signals between asymptomatic and symptomatic patients. Longitudinally, we note sleep and motor abnormalities that persisted for months after becoming COVID negative. Our study represents a novel phenotyping of the respiratory and behavioral trajectories of COVID recovery, and suggests that the two may be integral components of the COVID-19 syndrome. It further provides a proof-of-concept that contactless passive sensors may uniquely facilitate studying detailed longitudinal outcomes of COVID-19, particularly among older adults.

## Introduction

As the COVID-19 pandemic approaches the 2-year mark, a growing body of literature suggests that even after recovery from the acute viral illness, there may be a range of long-term neuropsychiatric sequelae (1–3). Further, the long-term outcomes of COVID-19 are affected by a broad range of factors including the presence of pre-existing medical or psychiatric conditions (4, 5), the nature and severity of the acute respiratory illness (6), the quality of care received in the short and long term (7), the patient's socioeconomic status (4, 8), and age (9). As a result, we see a very heterogeneous range of outcomes with COVID-19. The impact of this heterogeneity is especially pronounced in the context of behavior symptoms, which can manifest themselves as predisposing factors (10, 11), a core symptom of COVID-19 (12, 13), a virus-induced long-term symptom (2, 3), or an independent secondary effect (2, 14, 15).

These findings highlight the need for tracking the trajectory of COVID-19 disease through its acute and post-acute phase. Today, however, we lack solutions for collecting objective and longitudinal measurements from COVID-19 patients at home. Existing solutions for collecting data from patients at home rely mainly on self-reporting (9). Yet, self-reporting can be highly subjective, particularly when considering behavioral symptoms. Further, monitoring COVID-19 patients through the course of their disease is complicated by distancing and isolation protocols during the acute phase (16). It is also complicated by the difficulty of sustaining patient engagement in longitudinal post-acute studies (17, 18). While wearable devices and mobile phones may help collecting longitudinal data, such devices are ill-suited to older adults, and individuals who suffer from impaired memory and/or cognition (19).

**Figure 1:**
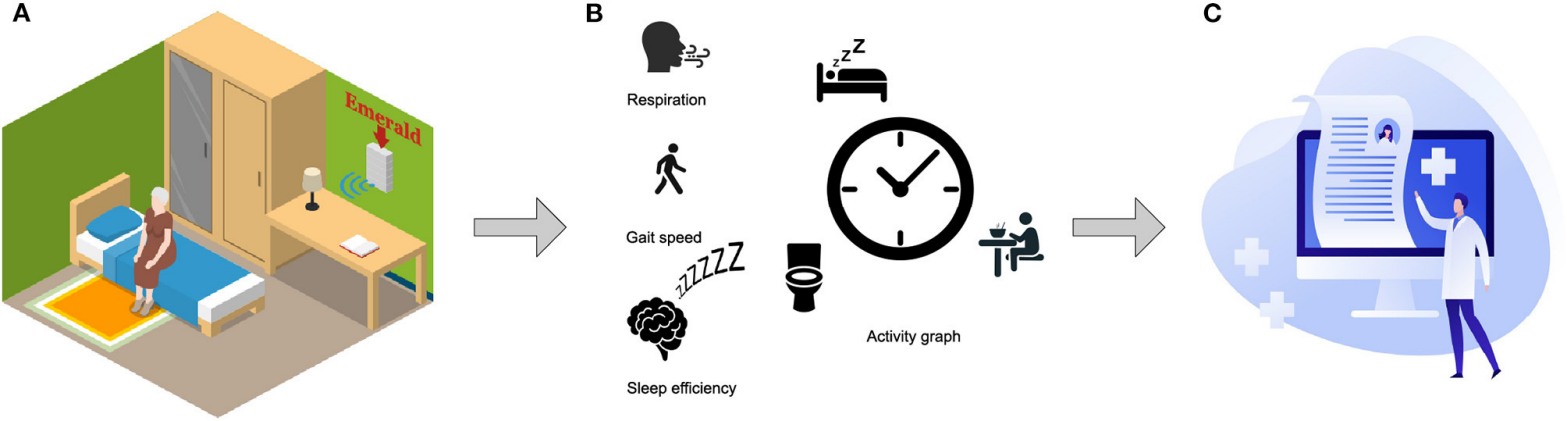
System overview. **(A)** The Emerald device sits in the background of the home like a Wi-Fi router. It sends out a wireless signal that is 1,000 times weaker than the home Wi-Fi, and analyses the reflected signal 24/7 without burdening the monitored subject. **(B)** Measurements of the trajectories of respiration, gait speed, sleep efficiency, and daily activities of the subject are generated automatically. **(C)** Clinicians and researchers (with the proper credentials) can access the data remotely at home or in the office.

We study a new solution for passively collecting objective and continuous measurements of COVID-19 patients recovering at home, during their active disease phase and post-acute recovery. We specifically focus on monitoring the trajectory of respiratory and behavioral phenotypes in older patients. We use a novel wireless sensor that sits in the background of the home (akin to a Wi-Fi router). The sensor, called Emerald, transmits very low power wireless signals, and analyzes their reflections from nearby humans and inanimate objects using machine learning (20, 21). It infers physiological and behavioral markers, including respiratory signals, gait speed, sleep patterns, and the time spent in different locations at home (activity graph). It can collect data continuously for prolonged periods, without physical contact with patients, and operates passively without burdening patients or caregivers. This sensor has been validated for monitoring sleep, gait speed, location, and respiration (20, 22, 23), and has been piloted in clinical studies of agitation (24), dementia (19), and Parkinson's disease (25). Figure 1 illustrates the operation of the Emerald sensor.

We present the results of 24/7 monitoring of three COVID-19 patients in an assisted living facility for 3 months. Our sensor technology offers a pragmatic solution to several of the challenges around long-term tracking in COVID-19, especially in older adults. It does not require any active engagement from the monitored person, and collects data passively and continuously, without exposing caregivers or others to the patient. We show how this approach facilitates continuous phenotyping of changes in physiological and behavioral parameters through the acute and post-acute phases of COVID-19.

Today's clinical studies are limited to episodic measurements, typically conducted in the clinic. In contrast, the approach described herein enables zooming in on individual patients in their natural living environment to obtain detailed and clinically meaningful measurements of their condition over long periods of time, and without interfering with patients' lives.

## Results

In total we monitored the subjects for 327 days. We processed the data to identify missing measurements due to accidental device unplugging, device malfunctioning, or patient being away in the hospital. After accounting for missing measurements, we collected 294 nights of sleep, 3,056 h of respiration signals, and 4,358 measurements of gait speed. A detailed description of subject recruitment and methods can be found in Supplementary Material.

### Clinical Course

In Table 1, we report the demographic and clinical characteristics of the study samples. Subject 1 is an 88yo male. His initial COVID symptoms were sore throat, fever and muscle ache. He was hospitalized for these symptoms and his monitoring began on day 0, upon return to the residential facility. He did not require any additional hospitalization. Subject 2 was an 81yo female with a pre-COVID history of COPD, generalized anxiety, and mild cognitive impairment. Her COVID symptoms started with fever, fatigue, and a sore throat. During the monitoring period, she was hospitalized due to breathing distress on day 7's early morning. She recovered gradually after discharge from hospital on day 14. Subject 3 is a 73yo female who tested positive for SARS Cov-2 infection but demonstrated no clinical symptoms of COVID throughout the study period. She had a prior history of bipolar disorder.

**Table 1:** Demographic and clinical characteristics of the study samples (*N* = 3).

**Variables**	**Subject 1**	**Subject 2**	**Subject 3**
Age	88	81	73
Sex	M	F	F
Monitoring begins	04/27/2020	04/07/2020	04/16/2020
Day confirmed COVID positive with respect to the first day of monitoring^*^	Day -11	Day -6	Day 0
Day confirmed COVID negative with respect to the first day of monitoring^*^	Day 8	Day 17	Day 8
Monitoring ends^*^	Day 106	Day 115	Day 106
Acute COVID-19 symptoms	•Fever •Fatigue •Sore throat •Muscle pain	•Fever •Fatigue •Sore throat	None
Other comorbid conditions	Obsessive compulsive disorder, Major depressive disorder	Mild cognitive impairment, Generalized anxiety disorder (GAD), Benzodiazepine abuse in remission, CHF, COPD	Major neurocognitive impairment (mixed type), Bipolar disorder

* *Days are relative to the first day of monitoring*.

### Respiratory Changes

**Figure 2:**
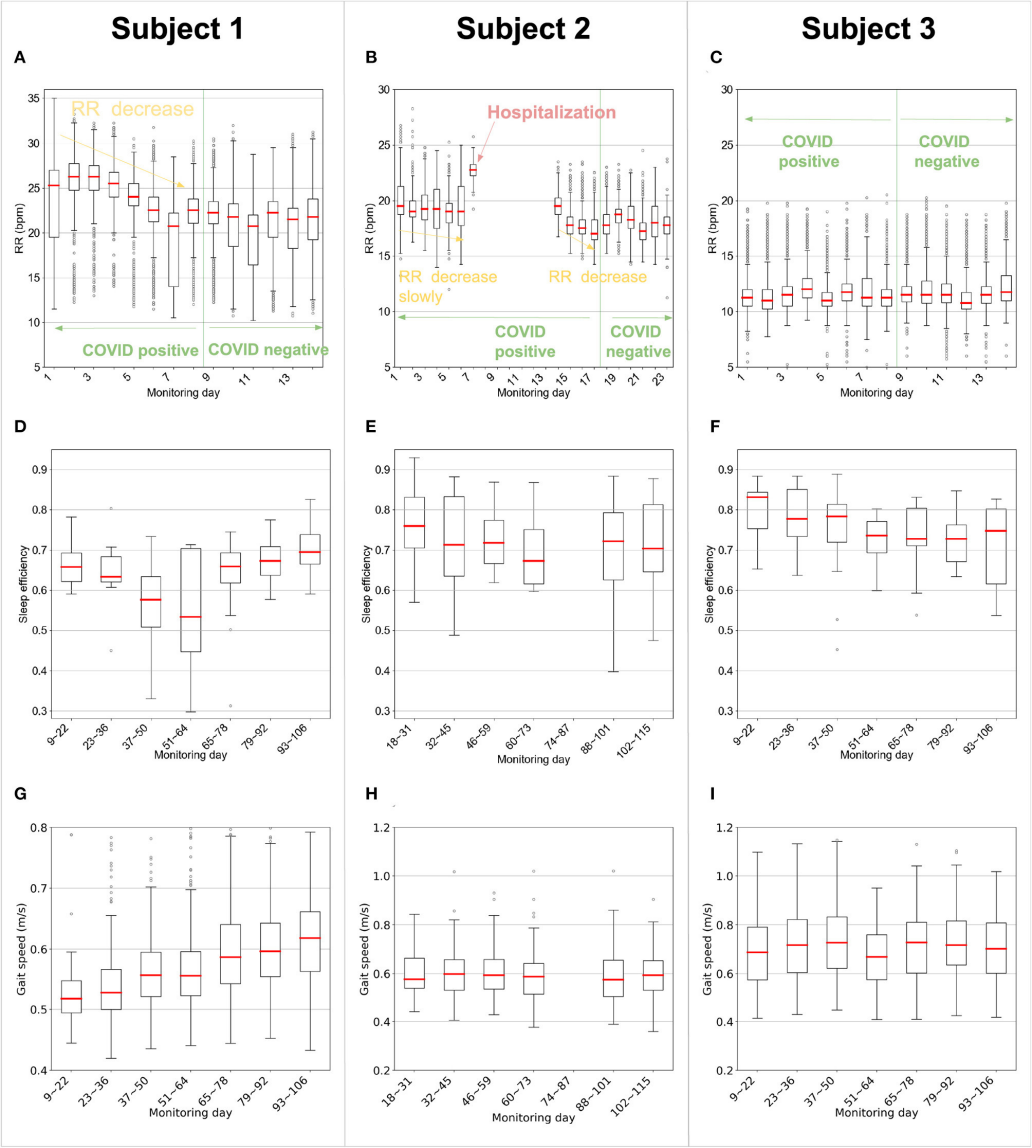
Contactless measurements of respiration rate, sleep efficiency, and gait speed. **(A–C)** The daily respiration rate (RR) of the patients during sleep as they recovered (for each boxplot sequentially, *n* = 962, 703, 964, 831, 766, 762, 863, 935, 1,193, 1,032, 998, 973, 1,218, and 1,137 for subject 1; *n* = 1,433, 1,003, 1,332, 1,393, 1,779, 1,694, 177, 1,059, 950, 1,077, 1,234, 1,001, 932, 1,715, 1,019, 1,403, and 783 for subject 2; *n* = 994, 928, 1,045, 1,012, 864, 815, 832, 820, 1,139, 920, 929, 851, 919, and 977 for subject 3). The figure shows that while being COVID positive, the symptomatic subjects experienced an elevated RR in comparison to their baselines. In contrast, the asymptomatic subject had an RR similar to her baseline. Further, the figure shows that prior to hospitalization, Subject 2 experienced an unusually elevated RR. **(D–F)** Sleep efficiency of the subjects computed as the ratio of the sleeping time to the time in bed. Each box refers to data for two consecutive weeks (for each boxplot sequentially, *n* = 14, 14, 14, 14, 14, 14 and 11 for subject 1; *n* = 14, 14, 14, 13, 12 and 12 for subject 2; *n* = 14, 14, 14, 14, 14, 12 and 9 for subject 3.). **(G–I)** Gait speed of the subjects, where each boxplot represents two consecutive weeks (for each boxplot sequentially, *n* = 40, 226, 309, 526, 356, 403, and 335 for subject 1; *n* = 58, 107, 133, 122, 136, and 126 for subject 2; *n* = 237, 123, 133, 94, 121, 174, and 183 for subject 3.). **(D–I)** From day 74–day 87, subject 2 has 12 days of data missing due to accidental unplugging of the device by the housing staff. There are only 2 days left for these 2 weeks, so the box is not plotted for this period. **(A–I)** In each box plot, the central line indicates the median, and the bottom and top edges of the box indicate the 25th and 75th percentiles, respectively. The whiskers extend to 1.5 times the interquartile range. Points beyond the whiskers are plotted individually using the circle symbol.

Figures 2A–C plots the daily respiratory rate (RR) of the three subjects, while being COVID positive, and as they transition to negative testing. The three subjects have different recovery experiences. The first two subjects are symptomatic, whereas subject 3 is asymptomatic. This is reflected in their RR in the figure, which shows that subjects 1 and 2 have significantly less stable RR than subject 3. Focusing on the symptomatic patients, subject 1 had a smooth gradual recovery, whereas subject 2 had respiration distress and was hospitalized for about a week. As shown in Figure 2A, subject 1 started with an elevated RR with a median of 25 breaths per minute (BPM) on day 1, and 26.3 BPM on day 2. Over the next 6 days, his median RR gradually decreased by about 3 BPM, and stabilized at a baseline around 21 BPM. In contrast, and as in Figure 2B, subject 2's RR initially decreased slowly to a median of 19 BPM; but on day 7, the RR suddenly jumped to 22 BPM. On that day the subject reported a medical emergency, was subsequently admitted to the hospital, and received medical treatment for breathing difficulty. After subject 2 came back from the hospital, her RR began to drop from a median of 19.5 BPM on day 14 to a baseline of 18.0 BPM on day 23. As for the asymptomatic patient, i.e., subject 3, her RR was stable for the whole recovery period with a daily median of 11.5 BPM, as shown in Figure 2C.

Next, we check whether the differences in RR for the symptomatic subjects are statistically meaningful. We divide the RR samples into two groups for each subject, where the first group refers to RR samples captured before becoming COVID negative and the second group refers to RR samples after the subject became COVID negative. We run a single-sided Mann–Whitney U test for each subject and calculate the effect size by Cohen's *d*. As expected, the RR elevation for the symptomatic subjects, i.e., subject 1 (*U* = 3.0E7, *p* = 1.8E-268) and subject 2 (*U* = 5.8E7, *p* = 2.6E-241), is statistically significant, but is insignificant for the asymptomatic subject 3 (U = 2.0E7, *p* = 0.9986). The Cohen's *d* for subject 1 (*d* = 0.52) and subject 2 (*d* = 0.53) shows medium effect size, while for subject 3 (*d* = −0.04) the effect size is minimum. Further, when comparing the hospitalization day of subject 2 (i.e., day 7) to days 1 to 6, we find the elevation is significant (single-sided Mann–Whitney *U* test, *U* = 1.2E6, *p* = 2.2E−90), and the effect size is large (Cohen's *d, d* = 2.0).

### Sleep Efficiency

Sleep efficiency is the ratio of the total sleep time to the total time in bed (26). Sleep efficiency is highly correlated with the mental status of an individual. Usually, the more anxious an individual is, the lower the sleep efficiency will be (27). Figures 2D–F reports the sleep efficiency for all 3 subjects. As shown in Figure 2D, subject 1 has low sleep efficiency that worsens around day 37 to 64 in the middle of the study but subsequently stabilizes. One-way ANOVA on these 7 groups in the figure verifies there is significant difference (*df* = 94; *F* = 15.57; *p* = 0.016) between the groups. More specifically, sleep efficiency from day 37~64 is significantly worse (single-sided *t*-test; *df* = 94; *t* = 3.85; *p* = 2.4E-4) than the other 10 weeks. This is consistent with the assisted living facility (ALF) staff's observations that subject 1 demonstrated some initial anxiety, but over time, his anxiety subsided, and he was able to recover. As shown in Figure 2E, subject 2's sleep efficiency decreased slightly over the monitoring period. Her sleep efficiency for the first 6 weeks is higher than the later 8 weeks (single-sided *t*-test; *df* = 78; *t* = 1.60; *p* = 0.057). Subject 3 had the best sleep efficiency overall (single-sided *t*-test; *df* = 264; *t* = 6.01; *p* = 3.7E-9); however, her sleep efficiency worsened from day 51 to day 106 (the end of the study) compared with day 9 to day 50 (single-sided *t*-test; *df* = 90; *t* = 2.69; *p* = 0.0044). This aligns with the ALF staff's observation that subject 3 has shown fewer health issues than the other two patients, though in the last 2 months of the study, she has experienced anxiety.

### Gait Speed

Figures 2G–I compares the trajectories of gait speed of the 3 participants. As shown in Figure 2G, subject 1 has a relatively low initial median walking speed of 0.52 m/s. However, his walking improves steadily in the following months to reach 0.62 m/s by the end of the monitoring period. Regression analysis shows that the rate of improvement in his gait speed was 0.0076 m/s per 7 days ([0.0068, 0.0085] 95% CI, *p* = 2.9E-66). The ALF staff reported that subject 1's walking was impaired initially, most likely due to his very limited mobility while in quarantine. Subsequently with physical therapy, his walking speed improved. Figure 2H shows that subject 2 did not exhibit a significant change in her gait speed over the course of the monitoring period (−0.0015 m/s per 7 days [−0.0035, 0.00037] 95% CI, *p* = 0.11). Similar to subject 2, subject 3's gait speed, in Figure 2I, is relatively steady during the observation period (−0.00076 m/s per 7 days [−0.0024, 0.00089] 95% CI, *p* = 0.37).

### Activity

**Figure 3:**
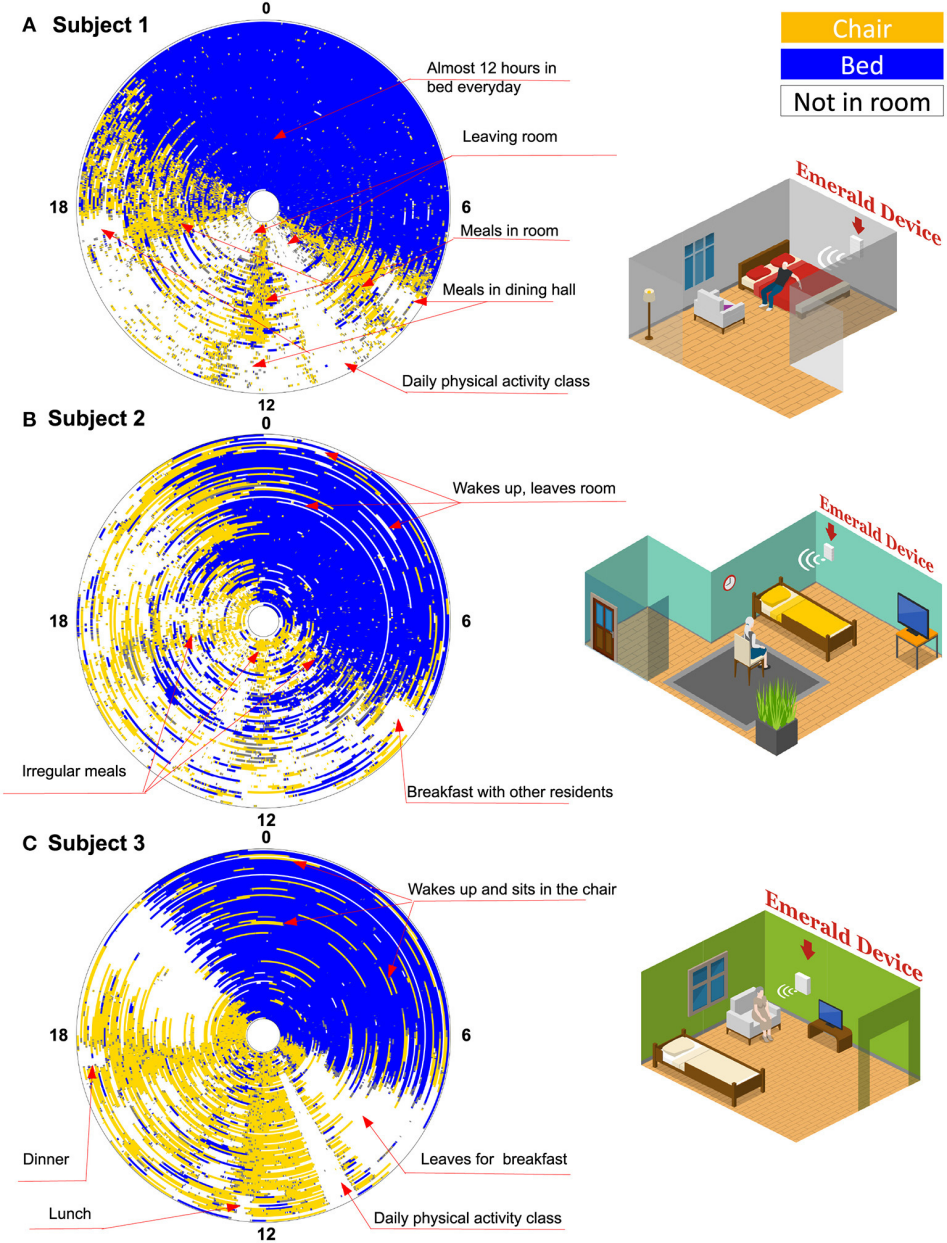
Activity graph. Each circle is 1 day. Angle around each circle represents the hour in the day (Zero at the top refers to midnight, and 12 refers to noon time). The innermost circle is the first day the patient became COVID negative, while the outermost circle is the last monitoring day for that patient. The different colors refer to different locations: bed in blue, chair in yellow, and outside the room in white. In total, there are 87 days for subject 1, 87 days for subject 2, and 99 days for subject 3 (Missing days are not visualized).

Figure 3 shows general behavior patterns for all three subjects. Each circle is one day (12 a.m. at the top refers to midnight, and 12 p.m. at the bottom refers to noon) the inner most circle is the first day after becoming COVID negative, and the outermost circle is the last day of monitoring. The graphs provide a longitudinal view of subjects' basic activities after they became COVID negative.

Based on Figure 3A, we note that subject 1 started to leave the room on the day he was declared COVID negative. This can be inferred from the white patches, which refer to time intervals during which the subject is outside the coverage of the Emerald wireless signals, i.e., outside his room at the ALF. The subject, however, continued to take his meals in his room rather than in the dining hall as indicated by the yellow cones (i.e., being on the chair) around 8 a.m., 12 p.m., and 5 p.m., in his activity graph. This continued until 29 days before the end of the study, when we see the yellow cones are replaced by white-colored region, which indicates that he started to take all of his meals in the dining room. He spent almost 12 h per day in his bed during the entire post-acute monitoring period. Subject 1 also left his room regularly around 10 a.m. every day, which coincides with daily physical activity classes (as confirmed by the ALF staff).

In Figure 3B, we note that subject 2 began to leave her room right after her quarantine period ended on day 17, as indicated by the white regions in her activity graph. Subject 2 seems to be in her room around mealtime; however, her eating patterns are not as regular as those of the other patients. This can be inferred from the lack of clear yellow cones (i.e., time on chair) around meal times, like those observed in the activity graphs of the other two patients. At the end of the study, subject 2 started leaving her room around breakfast time, which indicates that she started joining other residents for breakfast. During nighttime, it is relatively common for subject 2 to wake up in the middle of the night and leave her bed and the whole room as indicated by the white lines interspersed in the blue region. This behavior is confirmed with the staff at the ALF.

As shown in Figure 3C, subject 3 remained in her room for the first 2 weeks after lifting the quarantine. Later, she demonstrated a routine of leaving her room in the morning for breakfast then for a physical activity class. However, she remained in her room sitting on a chair for large parts of the remainder of the day and appeared to leave her room in the afternoon and evening (also for meals) only in the last few days that she was monitored. She has a regular diurnal cycle but it is relatively common for her to wake up at night and move from her bed to her chair (as indicated by the yellow lines in the blue region).

## Discussion

Today, clinical studies are limited to a few episodic measurements of each patient, which hampers their ability to track detailed longitudinal disease progression. Our primary aim was to conduct digital phenotyping of the acute and post-acute phases of COVID-19 using a novel radio sensor capable of simultaneously and continuously monitoring multiple physiological and behavioral parameters in a passive and contactless manner. Our results demonstrate that such an approach can facilitate remote tracking of changes in behavior and respiration, which in turn may be markers of the recovery process. The inherent properties of the Emerald device—i.e., eliminating the need for interaction with the device or active data entry on the part of patient, and no requirement for ongoing maintenance such as regular charging—have facilitated the collection of continuous longitudinal data.

Among our subjects, we noted that subject 2, who had the most significant symptoms from COVID-19, appeared to demonstrate the most severe longitudinal disruption of respiration, sleep and daily routine. While we cannot comment on which aspects may be related to COVID-19 vs. exacerbations of premorbid neuropsychiatric symptoms, subject 2's data point to the possibility that especially in older adults, there may be a relationship between behavior symptoms and the COVID-19 disorder itself. Subject 1 had a less severe initial manifestation of COVID-19. However, longitudinal phenotyping indicates a physiological consequence to prolonged quarantine (i.e., worsening mobility and changes to sleep). It is unclear whether this was a direct consequence of COVID-19 infection or born out of the strains of isolation. The data did, however, enable the staff to identify and promptly attend to these issues. In the case of subject 3 who was asymptomatic to COVID-19, phenotyping data shed light on how a combination of the virus itself and environmental strain from implementing social distancing within an assisted living facility may trigger changes in behavior, such as where to eat and routine on activities.

The study also sheds lights on the specific utility of the studied metrics. It shows that respiration rate may serve as a biomarker in tracking recovery status during the acute phase of the disease. For symptomatic subjects (i.e., subjects 1 and 2), the RR during the acute phase was elevated from its baseline, even when patients did not report breathing issues, and decreased to its baseline as patients became COVID negative. Further, an abnormally high RR is detected for subject 2 before her hospitalization, which indicates that a sudden elevation of RR could serve as a precursor to symptom escalation in COVID-19. In contrast, sleep and gait speed highlighted problems during the post-acute phase. Specifically, subject 1 developed movement difficulties and took several months to recover his mobility after becoming COVID negative. All subjects exhibited signs of sleep issues that persisted for a long time.

The activity graph is a novel matric that can objectively quantify subjects' daily behavior, and potential changes in their habits. In this graph, each day is abstracted and summarized in a single colored circle, allowing us to visualize daily and repetitive behavior. This provides novel insights into patients' quality of life and daily functions. For example, in our study, the activity graphs have revealed that, while all three subjects live in the same ALF and in principle follow the same ALF schedule, subjects 1 and 3 have regular routines, while subject 2 does not. This could be a sign of subject's 2 agitation and exacerbated cognitive impairment, which was repeatedly noted by the staff. The ability to capture subjects' routine provides a new metric that can help in behavioral phenotyping, an area that currently heavily depends on subjective questionnaires.

The study has several limitations. The sample size is small and may not be representative of the broader COVID-19 population. In addition, we were not able to collect objective behavioral data using standardized scales to compare sensor data with. Also, the monitored physiological signals are limited to respiration, sleep, walking speed and activities, and the monitored space is limited by the radio coverage area. Despite these limitations, we believe that the results demonstrate the feasibility of passive and contactless phenotyping, and its potential for studying the short- and long-term progression of COVID-related symptoms among older adults recovering at home. Our team aims to address these limitations, particularly the absence of comparison measures in ongoing and future work.

At the time of writing, there remains a lack of clarity on how the neuropsychiatric symptoms of COVID may play out in the long term. Our findings indicate that passive digital phenotyping can be a powerful tool to facilitate understanding this issue. With the ability to closely track intra-individual changes in respiration, gait, sleep, and activities, such an approach holds the potential to unlock relationships between different behavioral phenomena associated with COVID-19 infection and recovery. Additionally, contactless passive monitoring technologies can uniquely facilitate detailed longitudinal studies of symptoms' trajectories in older adults, without burdening patients or caregivers.

## Data Availability Statement

The raw data supporting the conclusions of this article will be made available by the authors, without undue reservation.

## Ethics Statement

The studies involving human participants were reviewed and approved by the MIT institutional review board and the Mass General Brigham Human Research Protections Program ceded review to the MIT IRB. The patients/participants provided their written informed consent to participate in this study.

## Author Contributions

IV and DK: initial study concept and design. GZ, IV, YL, WM, and DK: analysis and interpretation of data. WM: acquisition of data and device installation. RM, HC, and WM: human subject management. GZ, YL, and YY: Software/Code development. GZ and YL: statistical analysis. All authors contributed to writing the manuscript.

## Funding

The project was funded by the MIT J-Clinic for Machine Learning in Health, a research center at the Massachusetts Institute of Technology. IV was supported by the McLean Division for Geriatric Psychiatry.

## Conflict of Interest

DK is a co-founder of Emerald Innovations, which uses wireless sensors similar to the one used in the study to analyze physiological signals. The remaining authors declare that the research was conducted in the absence of any commercial or financial relationships that could be construed as a potential conflict of interest.

## Publisher's Note

All claims expressed in this article are solely those of the authors and do not necessarily represent those of their affiliated organizations, or those of the publisher, the editors and the reviewers. Any product that may be evaluated in this article, or claim that may be made by its manufacturer, is not guaranteed or endorsed by the publisher.
