# Supplementary material for: Contactless In-Home Monitoring of the Long-Term Respiratory and Behavioral Phenotypes in Older Adults With COVID-19: A Case Series
Source: Front Psychiatry. 2021 Oct 27;12:754169. doi: 10.3389/fpsyt.2021.754169 (PMC8580478; doi:10.3389/fpsyt.2021.754169)
Supplement: Supplementary file 1 [file Presentation_1.pdf]

Supplementary Material

1 METHODS AND MATERIALS

1.1 Subjects:

We deployed the Emerald sensor with three COVID-19 patients, and passively collected their physiological metrics. All subjects were residents at an assisted living facility (ALF) in Framingham, Massachusetts. Prior to the COVID-19 outbreak, our research team had conducted feasibility studies using the same technology to monitor behavior symptoms in patients with dementia (1,2). Thus, personnel at the facility were familiar with the technology and protocols. We recruited three residents who were infected by the SARS-CoV-2 in the spring of 2020. For all participants, we obtained consent either from the participant or authorized healthcare proxy shortly after their initial COVID positive test. This study was approved by the MIT institutional review board and the Mass General Brigham Human Research Protections Program ceded review to the MIT IRB.

1.2 Technology:

We use the Emerald device, which was developed at the MIT Computer Science and Artificial Intelligence Lab by members of our research team. The device is a Wi-Fi like box that transmits low power wireless signals and analyzes their reflections using machine learning algorithms. The Emerald device can localize patients and track their motion trajectories in a manner similar to radar. However, in contrast to radar, the transmission power of an Emerald radio is very low, about 1000 times lower than a Wi-Fi radio (3,4). Once installed, the device operates continuously without effort from patient or caregiver. The data is encrypted and stored in the cloud, so that it can be accessed and analyzed remotely with proper security credentials. Combining data from multiple devices in the home is feasible as shown in (5). However, for this COVID study, a single device was sufficient to cover the subject's unit.

1.3 Device deployment:

Device deployment was performed by the staff at the ALF. We built an iPhone app that allows a layperson to configure the device and connect it to the local Wi-Fi. The deployment followed simple steps: 1) Mount the device on the wall using 3M command strips (these are widely used strips to attach frames on walls). When mounting the device, the staff was asked to choose a location that is within a couple of meters from the bed and make the device face the bed. 2) Plug the device into the power outlet and wait until the green light turns on. 3) Open the iPhone app and wait until the app has found the device, then enter the Wi-Fi information to connect the device to the local Wi-Fi network.

1.4 Data collection and processing:

We first process the data to identify missing measurements. Missing measurements can be due to the device being unplugged, or the subject being absent from the room for the entire day (e.g., during hospitalization). Missing data can also occur due to Internet problems that prevent data uploading to the cloud, and device installation errors. For sleep analysis, if a night has missing data, we consider the whole night missing.

The Emerald algorithms have been validated for computing sleep metrics, gait speed, location, and respiration signal (3,4,6–8). We process the output of the system as follows.

1.4.1 Respiration:

We are able to extract the breathing signal while the person is relatively static, e.g., sitting or sleeping. The extracted breathing signal is a time series of normalized chest displacement similar to the signal produced by a breathing belt on the subject's chest. The respiration rate (RR) is then computed as the number of breaths per minute. The details of how respiration is extracted from radio signals are described in an earlier publications (7,9), which show that the average correlation between the extracted breathing signal and the respiration extracted from an FDA-approved breathing belt is 91% (9).

1.4.2 Sleep:

The sensor uses a deep neural network to infer a person's sleep stages from the radio signals that bounce off their body (8). For each period while the person is in bed, the device reports the sleep stages as wake, light sleep, deep sleep, and REM, at the granularity of 30-second epochs. We compute the sleep efficiency as the total sleep time divided by the time in bed. Prior publications have shown that the device can compute sleep efficiency accurately (average error is 1.8% and the standard deviation is 1.2%) (4).

1.4.3 Gait:

The device detects the subject's movements, and can capture their trajectories. We apply signal processing algorithms to extract the gait speed of the subject from movement trajectory data. We measure gait speed along paths that are relatively straight and extend for more than 2 meters. Prior publications have demonstrated the ability of this method to accurately measure an individual's position to within 15 cm, and their gait speed to within 0.025 m/s (3,6).

1.4.4 Activity:

The device is able to track location within a 30-foot radius from the device (6). Thus, we are able to monitor the patients' location, while they are in their unit in the ALF. We process the position of the patient over time with respect to the layout of the patient's unit to infer how the patient uses the space.

We also collected collateral information on the clinical progress and symptoms of each of our subjects from the ALF staff. This was done on an ad hoc basis via regularly scheduled meetings between the study team and facility staff. During meetings, we recorded notes on clinical progress and these were compared to device data to add clinical context.

1.5 Statistical analysis:

For breathing data, we run single-sided Mann–Whitney U tests to compare different breathing groups and calculate the effect size by Cohen's *d*. For sleep data, we use one-way ANOVA and single-sided t-test to compare different groups of sleep efficiency. We applied linear regression analysis for longitudinal gait speed data to determine whether there was significant improvement or decline across time. All levels of significance were set to $P < 0.05$. In all visualized box plots, the central line indicates the median, and the bottom and top edges of the box indicate the 25th and 75th percentiles. The

whiskers extend to 1.5 times the interquartile range. Points beyond the whiskers are plotted individually using the circle symbol.

2 Reference

1. Vahia IV, Kabelac Z, Hsu C-Y, Forester BP, Monette P, May R, Hobbs K, Munir U, Hoti K, Katabi D. Radio Signal Sensing and Signal Processing to Monitor Behavioral Symptoms in Dementia: A Case Study. *Am J Geriatr Psychiatry* (2020) 28:820–825. doi:10.1016/j.jagp.2020.02.012

2. Vahia IV, May R, Kabelac Z, Hoti K, Munir U, Owoyemi P, Monette P, Katabi D. AI-BASED DIGITAL PHENOTYPING OF BEHAVIORAL AND PSYCHIATRIC SYMPTOMS IN DEMENTIA: CLINICAL IMPLICATIONS. *Alzheimer's & Dementia* (2019) 15:P163. doi:10.1016/j.jalz.2019.06.4335

3. Hsu C-Y, Liu Y, Kabelac Z, Hristov R, Katabi D, Liu C. Extracting Gait Velocity and Stride Length from Surrounding Radio Signals. in *Proceedings of the 2017 CHI Conference on Human Factors in Computing Systems - CHI '17* (Denver, Colorado, USA: ACM Press), 2116–2126. doi:10.1145/3025453.3025937

4. Hsu C-Y, Ahuja A, Yue S, Hristov R, Kabelac Z, Katabi D. Zero-Effort In-Home Sleep and Insomnia Monitoring using Radio Signals. *Proc ACM Interact Mob Wearable Ubiquitous Technol* (2017) 1:1–18. doi:10.1145/3130924

5. Kabelac Z, Rahul H, Hristov R, Chan V, Hatch M, Han J, Cadavid D, Chang A, Katabi D. An In-Home Study of Facioscapulohumeral Muscular Dystrophy (FSHD) Patients using Contactless Wireless Sensing and Machine Learning (1561). *Neurology* (2020) 94: Available at: https://n.neurology.org/content/94/15_Supplement/1561 [Accessed September 21, 2021]

6. Adib F, Kabelac Z, Katabi D. Multi-person localization via {RF} body reflections. in *12th {USENIX} Symposium on Networked Systems Design and Implementation ({NSDI} 15)*, 279–292.

7. Adib F, Mao H, Kabelac Z, Katabi D, Miller RC. Smart Homes that Monitor Breathing and Heart Rate. in *Proceedings of the 33rd Annual ACM Conference on Human Factors in Computing Systems* (Seoul Republic of Korea: ACM), 837–846. doi:10.1145/2702123.2702200

8. Zhao M, Yue S, Katabi D, Jaakkola TS, Bianchi MT. Learning Sleep Stages from Radio Signals: A Conditional Adversarial Architecture. in *International Conference on Machine Learning* (PMLR), 4100–4109. Available at: http://proceedings.mlr.press/v70/zhao17d.html [Accessed April 12, 2021]

9. Yue S, He H, Wang H, Rahul H, Katabi D. Extracting Multi-Person Respiration from Entangled RF Signals. *Proc ACM Interact Mob Wearable Ubiquitous Technol* (2018) 2:86:1-86:22. doi:10.1145/3214289
